# Black GaAs: Gold-Assisted Chemical Etching for Light Trapping and Photon Recycling

**DOI:** 10.3390/mi11060573

**Published:** 2020-06-05

**Authors:** Paola Lova, Cesare Soci

**Affiliations:** School of Physical and Mathematical Sciences, Division of Physics and Applied Physics, Nanyang Technological University, 21 Nanyang Link, Singapore 637371, Singapore; csoci@ntu.edu.sg

**Keywords:** metal-assisted chemical etching, antireflection, *black GaAs*, photon recycling

## Abstract

Thanks to its excellent semiconductor properties, like high charge carrier mobility and absorption coefficient in the near infrared spectral region, GaAs is the material of choice for thin film photovoltaic devices. Because of its high reflectivity, surface microstructuring is a viable approach to further enhance photon absorption of GaAs and improve photovoltaic performance. To this end, metal-assisted chemical etching represents a simple, low-cost, and easy to scale-up microstructuring method, particularly when compared to dry etching methods. In this work, we show that the etched GaAs (*black GaAs*) has exceptional light trapping properties inducing a 120 times lower surface reflectance than that of polished GaAs and that the structured surface favors photon recycling. As a proof of principle, we investigate photon reabsorption in hybrid GaAs:poly (3-hexylthiophene) heterointerfaces.

## 1. Introduction

In the last decades several antireflective coatings, scattering layers and photon recycling structures have been developed to enhance photovoltaic performances in devices based on inorganic semiconductors, [1,2,3] donor-acceptor organic [4] and hybrid [5,6] systems, perovskites [7], and for light transmitting panels based on luminescent solar concentrators [8]. Photon absorption can also be enhanced using scattering and light trapping surfaces [9,10,11,12] or photonic crystals [13,14,15] that aim to increase the light optical path, to confine light in the active layer, or to recycle emitted photons [16]. Most of these structures are obtained by lithographic methods that produce high quality and homogeneous patterns, but often do not provide the high throughput necessary for the fabrication of large area devices. To this end, wet chemical methods are a viable, low-cost, and easy to scale-up alternative to lithographic patterning. Among these processes, metal-assisted chemical etching (MacEtch) became a paradigm for silicon structuring to produce surfaces with near-zero reflectance [17,18,19,20,21,22,23,24,25]. Etched black silicon is indeed a commercial product, and entered mass production of solar cells and modules [26,27]. The high throughput demonstrated for silicon made wet etching processes interesting also for translation to other semiconductors [28].

**Figure 1:**
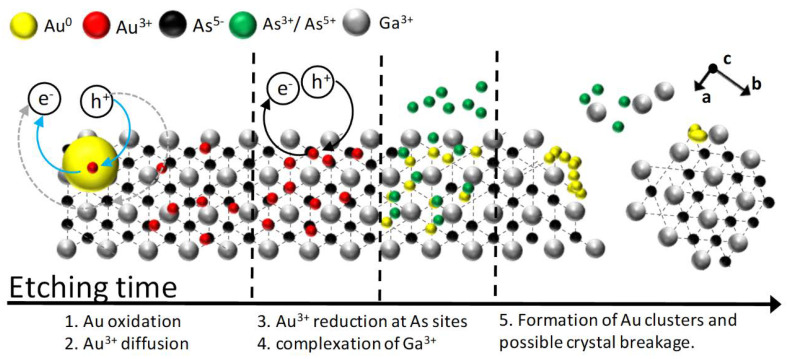
Schematic of the metal-assisted chemical etching (MacEtch) process of a GaAs (111)B surface.

MacEtch relies on the dissolution of a semiconductor surface catalyzed by a metal in a bath containing alkaline or acidic oxidizing agents [17,20,23,29,30,31]. The catalyst favors anisotropic substrate dissolution. Then, the substrate can be structured by patterned metal films [32,33] or etched randomly using dispersed metallic particles [24]. In recent years, several attempts aimed to extend MacEtch to III‒V group semiconductors [32,33,34,35,36,37,38,39,40,41,42,43], which yield better device characteristics in light emitting diodes and solar cells compared to mainstream silicon and germanium [44,45]. GaAs structuring via MacEtch has been reported in conjunction with catalyst vacuum depositions [46], or with metal patterning [32,33,34,35,36,37,38,39,40,41] by nanoimprint lithography [47], photolithography [20], and microsphere self-assembly [48]. While lithographic depositions allow highly controlled nanostructuring, they have limited room for scaling-up the fabrication. Recently, lithography-free MacEtch of GaAs was demonstrated by both electrode [49] and electrodeless deposition of gold nanoparticles [12]. In the first case, etching the (100) GaAs surface in a KMnO_4_:HF bath yields nanowire arrays similar to those reported for etched black silicon. This approach generates an effective medium with reflectance as low as 4% [49]. In the second case, etching in H_2_O_2_/HF baths induces crystal plane dependent dissolution rates and then light trapping surfaces with reflectance lower than 2%. [12] In this case, the crystal plane dependent etching rate is favored by the different reactivity of the Ga and As sp_3_ atoms [12]. A schematic of the etching reactions is illustrated in Figure 1. Specifically, a gold nanoparticle previously cast on the substrate is oxidized by the peroxide in the bath. The oxidized gold cations diffuse along the semiconductor surface and are selectively reduced at an arsenic site. Then, the precipitated catalyst can be further oxidized or form a electrochemical cell for gold deposition and surface etching [12,50]. Although catalyst ions diffusion and precipitation mechanism apparently contradict the etching mechanism reported in the seminal paper by Li and Bohn [17] and further confirmed by a number of researches [32], catalyst dissolution and redeposition have been demonstrated for two-step etching processes [50,51]. The selective auric reduction is favored by the higher reactivity of As with respect to Ga in the structure [52]. Indeed, the zinc-blende structure stabilizes trivalent Ga, and destabilizes As, which has valence five in electrophilic environment and an easy-to-oxidize pair of electrons. Arsenic is then oxidized to arsenic and arsenious acids, while Ga is complexed by fluorine anions allowing migration of the catalyst within the inner surface. Normally, monoatomic Ga planes, which only expose Ga atoms, are mildly reactive in the etchant solution. However, the catalytic effect of migrating gold ions is inhibited when a continuous catalyst film is cast on the semiconductor surface. In this case, the etching process does not depend on the crystal plane exposed to the bath [33,53,54,55,56,57].

As mentioned above, while lithographic patterning limits the scalability and throughput of the etching process, lithography-free MacEtch is a start-to-end solution process that is easy to scale up to wafer size. The etching of different GaAs crystallographic orientations was previously reported and light trapping properties were reported for etched GaAs (111)B and (100) surfaces [12]. In this work, we investigate the possibility to employ the etched structures for photon recycling in hybrid polymer-inorganic heterointerfaces. Reabsorption of emitted photons is indeed particularly desirable in hybrid photovoltaic devices which suffer of low power conversion efficiency related to high charges recombination and low charge carrier mobility [58,59,60]. The possibility to reabsorb photons emitted upon charge recombination, which would otherwise increase losses, could improve the performance of these devices [58,59,60]. It was indeed demonstrated that charge generation at hybrid heterointerfaces occurs in both the polymer and in the inorganic material [6]. Photon reabsorption, therefore, could lead to larger generation yield in hybrid devices. To assess the possibility to employ the process for the fabrication of photon recycling surfaces, as a proof of principle, we investigate the photoluminescence reabsorption occurring at the heterointerface between structured GaAs and poly(3-hexiltiphene) (P3HT), which is a widely studied conjugated polymer in hybrid and tandem photovoltaic devices.

## 2. Materials and Methods

**Metal-assisted chemical etching**: the MacEtch was performed at room temperature on *n*-type (100), (110), (111)B, and (211) epi-ready GaAs wafers (Axt Inc, Fremont, CA, USA). In the process, gold nanoparticles were first deposited on the GaAs surfaces by immersion in a water solution containing 0.1 mM of AuCl_3_ (Sigma Aldrich, Saint Louis, MO, USA). The samples were successively blow-dried and etched in a bath of HF and H_2_O_2_ (4:1) in a Teflon^®^ container (DuPont Wilmington, DE, United States) for about 10 min under vigorous stirring. The samples were then removed from the bath, rinsed in deionized water, and finally blow-dried. Au nanoparticles removal was performed by further etching of the (111)B microstructured surface in aqua regia (HNO_3_/HCl = 1:3) with different dilution in water (not diluted, 1:5, and 1:10).

**P3HT deposition:** P3HT was deposited on quartz substrates (Hellma Müllheim, Baden Württemberg, Germany) and on polished and etched GaAs by spin coating a 10% (*w*/*v*) solution in dichlorobenzene at a rotation speed of 800 rpm. All the samples were annealed for 15 min at 120 °C on a hotplate (VWR, Radnor, PA, United States). Deposition and annealing were performed in nitrogen environment to avoid polymer oxidation.

**Structural and optical characterization:** Gold nanoparticles and microstructure images of the etched GaAs samples were collected by scanning electron microscopy (SEM) with a field emission Jeol JSM-6700F (Jeol, Akishima, Japan) endowed with a secondary electron detector. Particle size distribution was retrieved manually. Representations of the GaAs crystal lattice were elaborated with the software VESTA (JP-Minerals, Ibaraki, Japan) [61] using data retrieved from the Crystallography Open Database [62]. Normal incidence reflectance was collected with a Bruker Vertex 80v Fourier transform spectrometer (Bruker, Billica, MA, USA) coupled with a Bruker HYPERION microscope (Bruker). Photoluminescence spectra were collected at room temperature using a Horiba Fluorolog spectrofluorometer (Horiba, Kyoto, Japan) with a CCD detector, a Xenon lamp as the exciting source, and a monochromator to select excitation wavelength. The measurements were collected exciting both P3HT and GaAs at 500 nm.

## 3. Results and Discussion

**Figure 2:**
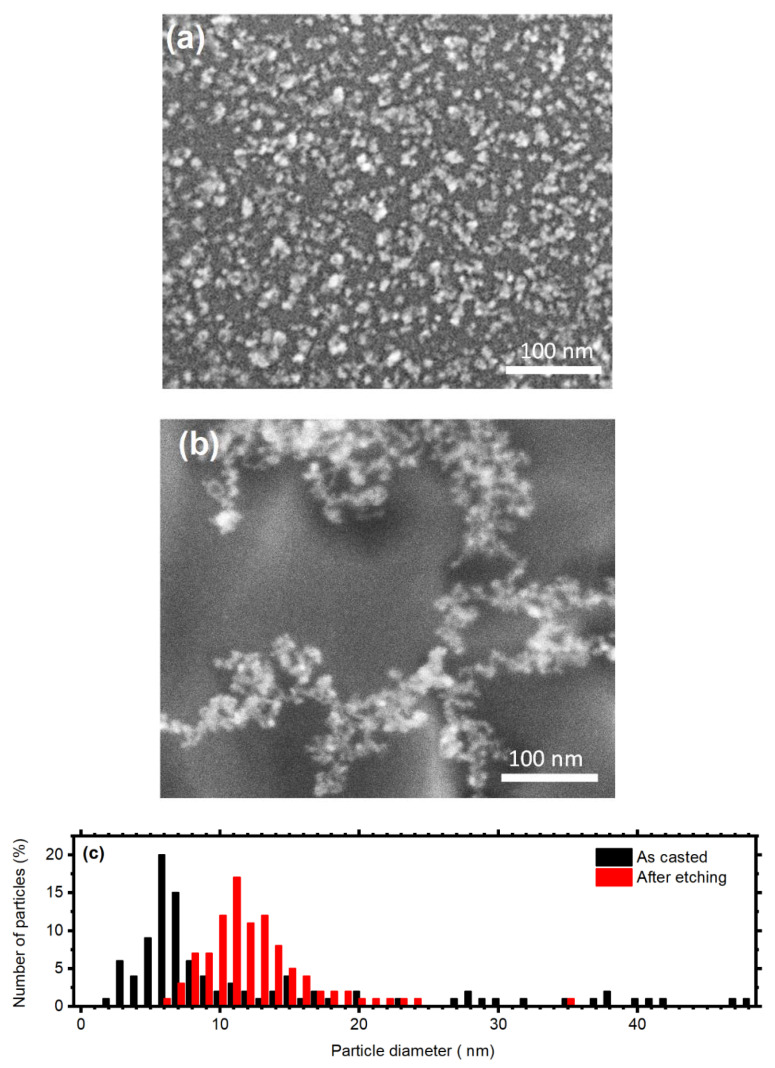
SEM micrographs showing Au nanoparticles on the GaAs surface as casted (**a**) and after the etching process. (**c**) Particles size distribution before (black) and after (**b**) GaAs MacEtch.

**Figure 3:**
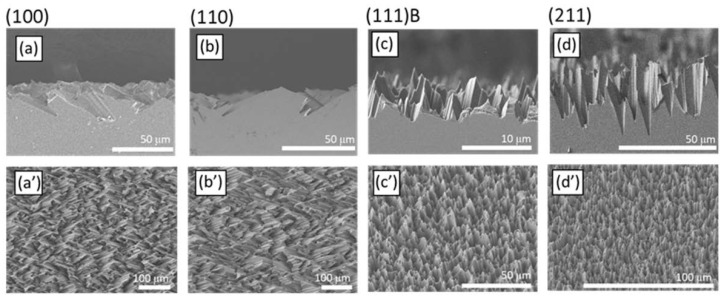

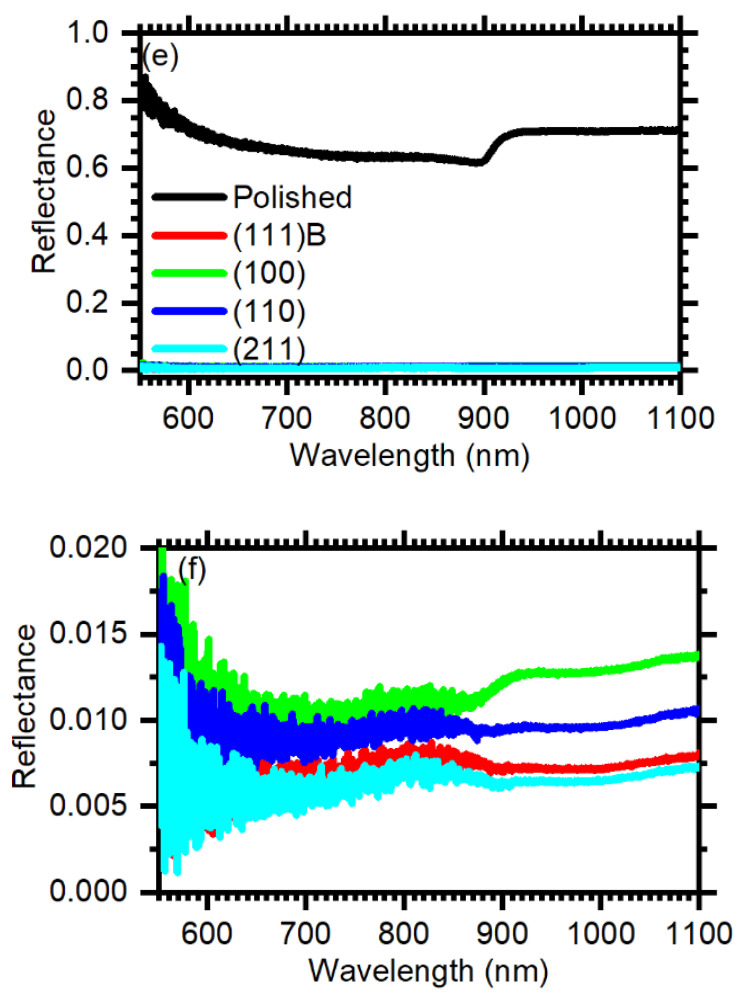
Cross-sectional (**a**–**d**) and tilted view (**a’**–**d’**) SEM micrographs of the (100) (**a**,**a’**), (110) (**b**,**b’**), (111)B (**c**,**c’**), and (211) (**d**,**d’**) GaAs crystalline planes. (**e**) Normal incidence reflectance spectra of polished (black) and etched GaAs surfaces: (100), red; (110), green; (111)B, blue; (211), cyan. (**f**) Reflectance spectra of the etched samples: A magnification of the reflectance scale allows to appreciate the spectral features.

**Figure 4:**
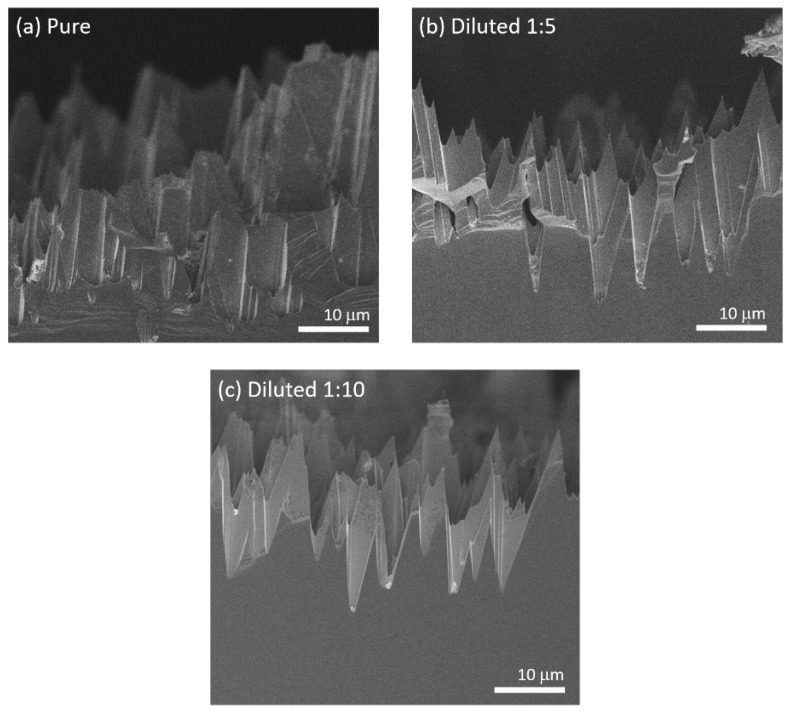
Cross-sectional SEM micrographs of the (111)B etched surface after 30 min treatment in pure aqua regia (**a**), and in aqua regia diluted in water with proportion 1:5 (**b**) and 1:10 (**c**).

The effective electrodeless deposition of Au nanoparticles on the GaAs surface was confirmed by SEM measurements. Figure 2a reports a micrograph of the pristine particles, which are distributed densely and homogeneously on the GaAs surface. The diameter distribution shown in Figure 2c in black color displays that the catalyst has a broad size dispersion, whit diameters ranging from 3 nm to about 50 nm, while the larger fraction has a diameter approaching 7 nm. These data suggest the presence of particles aggregates on the semiconductor surface, which are not easily recognizable form single particles owing to the instrumental resolution. Figure 2b reports instead a SEM micrograph showing the gold particles on the surface after the etching process. In this case, they are not homogeneously distributed over the sample. Indeed after the etching, the catalysts are located within the etched features (see Figure 3 and Figure 4). However, the particles dimension is more homogenous with respect to the previous case. The size dispersion shown in red in Figure 2c indicates a sharper distribution with diameters ranging from 6 to 35 nm, with the larger percentage having a diameter equal to 11 nm. The variations in the particles size and distribution can be attributed to the continuous gold oxidation and reduction, which implies its dissolution and reprecipitation may decrease the diameter polydispersity.

Figure 3 shows the SEM micrographs collected after etching of the (100) (panels a, a’), (110) (panels b, b’), (111)B (panels c, c’), and (211) (panels d, d’) surfaces. Similar results were discussed in a previous work [12], but the brief analysis presented here provides a better interpretation of photon recycling mechanism of the etched surface. The top panels (a–d) of the figure display the cross-sectional micrographs collected for the different crystalline planes, while the bottom panels (a’–d’) show a tilted view at lower magnification. Etching of the (100) GaAs crystalline plane provides facets with ca. 25° characteristic angle with the sample plane and size of about 30 μm (Figure 3a). While these facets are partly exposed on the sample surface, the micrograph also shows the formation of holes beneath the surface. Figure 3a’ shows that these facets cover a large area, giving a homogeneous appearance to the surface. Figure 3b,b’ displays the results for the (110) plane. It is clear from Figure 3b that the surface exposes facets with similar orientation and size than those observed for the (100) surface. Panel b’ shows again a homogeneous distribution of etched planes on the entire sample surface. In the case of the (111)B surface, the etching generates randomly oriented hillocks (Figure 3c) and a homogenous surface (Figure 3c’). The facets on the etched (111)B orientation form larger angles than those observed in the (100) and (110) planes. Etching of the (211) plane digs conic-like holes into the surface and some spikes. These structures form again characteristic angles with respect to the sample plane (Figure 3d). Additionally, in this case, the resulting microstructures are homogeneously distributed and appear similar to those formed on the (111)B surface (Figure 3d’). Comparing the features size, it is possible to notice that the (111) surface provides less deep features than those arising from (211), (100), and (110) ones, which are instead similar in size. We suggest that the three surfaces showing deeper features are indeed etched at a faster rate, as monoatomic low-rate gallium planes are exposed to the etchant only at a later stage (i.e., the visible features in Figure 3a,b,d). Conversely, the etching of the highly reactive (111)B plane exposes the underlying (111)A plane (see Figure 1) where gallium atoms with mild reactivity can slow down the process.

The optical characteristics of the samples can be understood in terms of geometrical light trapping. The feature size, of the order of a few micrometers, is too large relative to visible and near infrared wavelengths to be treated within the effective medium theory. Light is therefore trapped within the etched surfaces upon multiple specular reflections. Figure 3e compares the reflectance spectra of the four etched samples with the one of a polished GaAs surface. The reflectance of polished GaAs approaches 0.7 in the range between 1100 and 925 nm. Moving toward shorter wavelengths, the values decreases to about 0.6 below 925 nm and remains rather constant until 700 nm where it increases and reaches ~0.85 at 550 nm. Conversely, the reflectance of etched GaAs approaches zero in the entire spectral range. The spectra are better visible in Figure 3f, where the intensity scale has been expanded. Within the GaAs transparency region, the minimum reflectance values are 0.013, 0.01, 0.008, and 0.007 for the (100), (110), (111)B, and (211) surfaces, respectively. Therefore, in the best case, MacEtch induces a 120-fold reduction of light reflection for the (211) surface at 550 nm (0.85/0.007).

Because the presence of residual gold would be detrimental for devices, we tested the possibility to dissolve the particles through a second etching step in aqua regia. Figure 4a reports the cross-sectional micrograph of the (111)B surface after the two MacEtch processes (see experimental section). There, gold is barely visible in the sample. However, several features on the surface appears broken. To reduce this detrimental effect, we also tested diluted etchants. Figure 4b shows the micrograph of a sample after etching with the acidic solution diluted 1:5 with water. Also in this case gold is barely visible, while the damages to the features are less evident than in the previous case. After further diluting the solution (1:10), the features are not damaged at all, but gold nanoparticles are still clearly visible in the micrograph as bright spots at the base of the etched features (Figure 4c). Then, we can infer that the dilution 1:5 can successfully remove the gold particles at a low expense for the microstructure quality.

**Figure 5:**
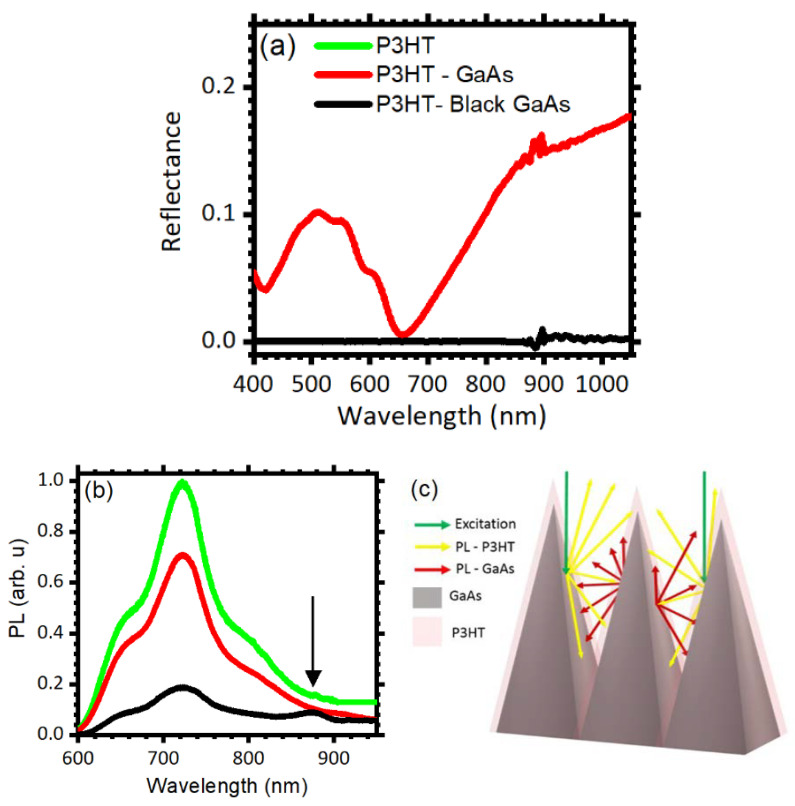
(**a**) Reflectance spectra collected for polished (red) and etched (black) GaAs (111)B surfaces covered with a P3HT thin layer. (**b**) Photoluminescence spectra collected for a P3HT film cast on a quartz substrate (green line), on polished GaAs (red line), and on *black GaAs* (black line). (**c**) Scheme of photon recycling at the *black GaAs*–P3HT interfaces.

As mentioned earlier, light trapping is a promising mechanism for photon recycling in hybrid polymer-inorganic optoelectronic devices. To demonstrate that the etched GaAs favors reabsorption of emitted photons, as a proof of principle, we cast a thin film of P3HT on the etched (111)B surface and investigated the resulting optical properties of the hybrid system. Figure 5a compares the reflectance spectra of the bare *black GaAs* (black line) with the one collected for *black GaAs* covered with P3HT (red line). The presence of P3HT increases the overall reflectance. While the bare *black GaAs* reflectance approaches zero in the entire spectral range, addition of the P3HT film generates a structured spectrum. The data show a maximum at 1100 nm, where the reflectance approaches 0.15. Moving toward shorter wavelengths, the intensity decreases slowly until ca. 870 nm. At energies above the GaAs energy gap, the reflectance then decreases with a faster rate until 660 nm, where it approaches zero. On the short wavelength side, a broad peak is detected between 420 and 660 nm, with a maximum at 505 nm and structures at 470, 570, and 611 nm, which are assigned to P3HT [6,63,64,65]. Overall, these data confirm the effective coverage of the surface by the polymer. To assess the effectiveness of photon recycling, we excited both P3HT and GaAs at 500 nm and compared the emission spectra of flat and etched surfaces. Photon recycling is expected to yield reduction of the P3HT photoluminescence and enhancement of the GaAs photoluminescence signals upon photon reabsorption in GaAs, favored by the trapping geometry. Figure 5b compares the emission spectrum collected for bare P3HT (green line) with P3HT cast on polished (red line) and *black GaAs* (black line). The bare P3HT emission spectrum ranges between 600 nm and 900 nm with a maximum of intensity at ~720 nm (green line) [66,67]. In the polished GaAs/P3HT spectrum no other features than those assigned to P3HT are evident and the emission intensity is lowered, suggesting electron transfer at the P3HT:GaAs interface (red line) [6]. The *black GaAs*/P3HT sample is characterized by stronger P3HT photoluminescence reduction compared to the flat sample, and by the presence of a peak at ~875 nm assigned to GaAs emission (black line) [12,33,68,69]. Quenching of the P3HT emission in the etched sample is consistent with geometrical light trapping discussed previously. Indeed, as sketched in Figure 5c, photons emitted from P3HT undergo several reflections between the surface features before they can leave the system, favoring reabsorption in GaAs. Because P3HT emission occurs below the HOMO‒LUMO transition of the polymer, emitted photons cannot be reabsorbed by P3HT and therefore excite the underlying GaAs.

These results are an important demonstration of a possible application of *black GaAs* as a photoactive material in hybrid solar cells. The superior semiconducting properties of GaAs, together with efficient light trapping and reabsorption of emitted photons shown here, could be employed to devise various hybrid structures for high performance photovoltaic devices.

## 4. Conclusions

We demonstrated that microstructured GaAs surfaces provide an efficient photon recycling platform to reduce losses associated with light emission occurring upon charge recombination in hybrid polymer-inorganic heterointerfaces. The microstructures were obtained by wet chemical etching of different GaAs crystalline planes in HF/H_2_O_2_, catalyzed by gold nanoparticles dispersed randomly on the semiconductor by electrodeless deposition. The process favors suppression of light reflection from the GaAs surface, with up to a 120-fold reduction. These are the best antireflective properties achieved for wet-etched semiconductors. Investigation of photoluminescence properties of GaAs:P3HT heterointerfaces confirmed that the microstructured GaAs reabsorb photons emitted by the P3HT cast on its surface. On the whole, the MacEtch process could be added to the fabrication workflow of photodetectors and solar cell devices to further improve their characteristics.
